# Editorial: Bio-based solutions for sustainable development of agriculture

**DOI:** 10.3389/fpls.2022.1056140

**Published:** 2022-10-17

**Authors:** Eduardo V. Soares, Spyridon A. Petropoulos, Helena M. V. M. Soares

**Affiliations:** ^1^Bioengineering Laboratory, Instituto Superior de Engenharia do Porto (ISEP)-School of Engineering, Polytechnic Institute of Porto, Porto, Portugal; ^2^Centro de Engenharia Biológica (CEB)−Centre of Biological Engineering, University of Minho, Braga, Portugal; ^3^Laboratório Associado em Biotecnologia, Bioengenharia e Sistemas Eletromecânicos (LABBELS) – Associate Laboratory, Braga−Guimarães, Portugal; ^4^Department of Agriculture Crop Production and Rural Environment, University of Thessaly, Volos, Greece; ^5^Rede de Química e Tecnologia (REQUIMTE)/Laboratório Associado para a Química Verde (LAQV), Departamento de Engenharia Química, Faculdade de Engenharia, Universidade do Porto, Porto, Portugal

**Keywords:** biofertilizers, biostimulants, environmental stress, inhibition of phytopathogens (biocontrol), nutrient use efficiency, mycorrhizal fungi, microbial inoculants, microbial siderophores

The modern agricultural sector is facing major challenges related to the increase of global population and food security that threatens many regions of the world. Considering that we expect an increase by 20% of global population until 2050, the agricultural industry has to cope with the increasing demands for food. However, despite the rapid evolution of cropping techniques and farming systems that have substantially improved crop performance over the years, the ongoing climate crisis, the ever-expanding anthropogenic activities and the irrational use of natural resources has put new obstacles since plants have to grow under unfavourable conditions, which severely affect the yield and quality of the marketable product.

Therefore, modern science has to provide new pillars to support the farming sector and identify alternative and sustainable agronomic practices and shift from agrochemicals to bio-based solutions to confront the current and emerging challenges (Ferreira et al., 2019a; Ferreira et al., 2019b; Petropoulos, 2020; Soares, 2022). In this context, Liu et al. evaluated the use of green manuring as an alternative practice to chemical control of goosegrass (*Eleusine indica* L.) in paddy fields. For this purpose, the authors tested a very common species for green manuring, namely milk vetch (*Astragalus sinicus* L.), in a series of experiments including the use of aqueous extracts, decomposed liquid material containing milk vetch powder and, finally, pot experiments where milk vetch powder was added in the growing medium. The obtained results showed great potential for incorporating green manure of milk vetch in soil as a sustainable weed control practice. The biocontrol of phytopathogens was examined by Minchev et al. who designed two synthetic microbial communities (SynComs), composed of bacteria (including, *Bacillus* spp. and *Pseudomonas* spp.) and fungi (*Trichoderma* spp. and *Rhizophagus irregularis*) with complementary biocontrol modes of action. The efficacy of the consortia to control shoot (*Botrytis cinerea*) and root (*Fusarium oxysporum*) pathogens was compared. The results showed that microbial consortia (SynComs) are more versatile (widest protection) than individual microbial inoculants, as they allow the efficient and simultaneous control of both pathogens, under different application methods. In the same line, Soudani et al. studied the tolerance of tomato induced by essential oil produced by *Artemisia absinthium* (AEO), against phytopathogens. A protective effect on tomato seedlings, grown under hydroponics conditions after previous treatment of the seeds with AEO, against *Fusarium oxysporum* sp. *oxysporum radicis lycopersici* (*Fol*) was observed together with: the decrease of various plant disease effects (e.g. fresh weight loss, chlorosis and tissue necrosis); the increase of plant defenses (reactive oxygen species production); callose deposition on seeds surface, and metabolomics and transcriptomics induction modifications that all together resulted in a long term tolerance against the tested fungus. On the other hand, Tran et al. evaluated the efficacy of rhizobacterial strains of *Streptomyces* against *Fusarium verticillioides*, the causal agent of the disease in maize crop known as Fusarium ear rot. The authors showed that *Streptomyces* spp. presented two modes of action against *F. verticillioides.* A direct antagonism, observed *in vitro* and *in vivo* studies, which is characterized by the inhibition of fungal growth and the repression of the production of the mycotoxin fumonisin, as well as the transient modification of expression profiles of the genes *AUX1*, *ARF1*, and *ARF2* (associated with biosynthesis pathway of auxin), and *AN1* (a gibberellic acid-related gene). In addition, a slight induction of the expression of genes associated with salicylic and jasmonic acid biosynthesis and pathogenesis-related proteins was recorded. Moreover, *Streptomyces* spp. also promoted plant growth even when infected with the fungus. Entomopathogenic fungi (EPF), besides their classical action as insect killers, can also perform other functions, such as colonizing various host plants (endophytic fungi), antagonizing phytopathogens and promoting plant growth (biofertilizers), which opens a new opportunity for its use in a sustainable agriculture. Bamisile et al. reviewed the mechanisms involved in plant growth promotion and defence against diseases by endophytic EPF and presented the current knowledge, challenges and limitations associated with the use of these fungi as an alternative to classical agrochemicals of synthetic origin.

In the study of Wang et al., the biostimulatory activity of zaxinone mimics was tested in three vegetable crops, namely tomato, pepper and squash grown under saline conditions. The obtained results showed that the specific compound increased plant growth and yield parameters (fruit size and total fruit weight) when applied at low concentrations (3 μM), while crop performance was higher for zaxinone mimics compared to humic acid, which is commonly used as biostimulant. De Paula et al. evaluated the effect of endophytic and rhizospheric plant growth promoting bacteria (PGPB) on a forage crop, namely *Paspalum atratum*, under *in vivo* and *in vitro* conditions. The *in vitro* tests allowed the isolation of 116 strains of bacteria, while 43 of them showed positive results in regards to nitrogen fixation, phosphate solubilisation and indole acetic acid biosynthesis. Further *in vivo* studies with 8 selected strains indicated 3 strains that significantly improved plant growth of *P. atratum.* The effect of plant growth promoting bacteria, as well as arbuscular mycorrhiza fungi (AMF) was also tested on soybean plants by Ngosong et al. aiming to evaluate its effects under nutrients restriction. The authors suggested that inoculation with PGPB and/or AMF significantly increased root nodulation and acid phosphatase activities, while they also increased the number of effective root nodules resulting in higher crop yield and grain content in macronutrients (carbohydrates, proteins) and minerals (Fe and Zn). However, they also noted that these effects were more profound when PGPB and AMF were integrated with mineral fertilizers. Another strategy, developed by Yadav et al. was based on the inoculation of four plant growth-promoting rhizobacteria, isolated from the rhizosphere of chickpea, to solubilize zinc from the rhizosphere of wheat. As a consequence, a concomitant improvement of wheat growth was observed together with an increase of zinc content in the grains and an over-expression of several TaZIP transport genes in the roots. Enriching the Fe content of plants is an important aspect both for the purpose of increasing crop productivity and for overcoming iron deficiency (the main form of micronutrients malnutrition) and, thus, improving human health. Lurthy et al. reviewed the impact of rhizosphere microbiota (highlighting the influence of plant-microorganism interaction) on increasing the iron content (biofortification) of comestible plant parts. The authors proposed a more holistic approach, based on the characteristics of plants and microorganisms, in order a better assimilation of Fe by plants can be achieved. The efficacy of a fertilizer resultant from a nitrified liquid anaerobic digestate supplemented with mineral nutrients (P, S and/or B) was evaluated by Weimers et al. for the production of leafy vegetables grown on peat-based growing media and compared to standard mineral fertilizers. Results demonstrated that plants fertilized with the non-supplemented mineral digestate evidenced S and B deficiency and early P deficiency, whereas plants fertilized with the supplemented mineral digestate resulted in sufficient plant tissue contents for all elements (except for S) and similar marketable yields comparatively to those fertilized with standard mineral fertilizer. A two-year field experiment study was performed by Yang et al. where the effect of various combinations of organic fertilizers (OF; corresponding to 30%, 50% and 70% of the total nitrogen applied) were assessed for improving the wheat yields with simultaneous minimization of nitrogen leaching and compared with the controlled-release urea (CU) effect. Whereas CU fertilization resulted in a rapid release of nitrogen in the first two months, a slow release of nitrogen combined with a significant residual effect was observed for the OF fertilizers combinations. Moreover, superior grain yield and nitrogen uptake was observed using two fertilizer combinations (30%OF+70%CU and 50%OF+50%CU) comparatively to the urea treatment.

Chen et al. evaluated the physiological and mechanistic functions of arginine on the growth of apple (*Malus hupehensis*) under nitrogen deficiency aiming to increase the tolerance of plants to nitrogen deficiency and, thus, to reduce the use of nitrogenous fertilizers. Under low nitrogen stress, external arginine supplementation, besides providing nitrogen to plants, also promoted the absorption and use of phosphorus, nitrogen and potassium, increasing the overall plant photosynthetic capacity and the antioxidant capacity of the plants. Modifications in the synthesis and metabolism of amino acids (namely, glutamate and ornithine) were also observed and they were reflected in the urea and Krebs cycles. Singh et al. examined the potential of water-stress alleviating effects obtained from biochar incorporation into the soil on maize crop. The authors tested two biochar materials (hardwood and softwood) and three irrigation levels [100%, 70% and 40% of evapotranspiration (ETc)] and suggested that hardwood affected soil properties (bulk density and soil porosity), while moderate water stress (70% ETc) recorded similar values to the control treatment (100% ETc) for the evaluated growth parameters and crop performance. On the other hand, Li et al. evaluated the importance of the PGPB rhizobacteria *Rahnella aquatili*s JZ-GX1 in promoting salt tolerance of the plant *Robinia pseudoacacia.* It was shown that plant seedlings, under salt stress (100 mM NaCl), exposed (through the roots) to bacterium presented an increase in fresh weight and root development compared to untreated ones. The exposure of plant seedlings to volatile organic compounds, produced by the bacterium, led to a reduction in oxidative stress indicators and an increase in enzymatic antioxidant defences and proline (an osmoprotectant) content in plant leaves. The authors proposed that 2,3-butanediol can be one of the relevant signalling compounds in the enhancement of salt tolerance in plants. In their work, Ahmad et al. started by revising the impact caused by heat stress on plants at various plant levels (cellular, organellar and plant as a whole) during different stages of plant growth as well as their natural tolerance mechanisms to combat it. Subsequently, the role of PGPB on the mediation of thermotolerance in plants was highlighted, emphasizing their role on the regulation of various enzymes, phytohormones and metabolites that contribute to induce heat tolerance in plants.

In summary, this Research Topic comprises a collection of 16 articles that offer new and updated knowledge about biofertilizers, biocontrol and improved resilience to environmental stressors. The information presented can be useful in the future development of bio-based products that are expected to be used, as an alternative to current agrochemicals, in modern and more sustainable agriculture.

## Author contributions

All authors have made a substantial, direct and intellectual contribution to the work, and approved it for publication.

## Funding

ES and HS are grateful to the Portuguese Foundation for Science and Technology (FCT) for financial support, funded by national funds through the FCT/MCTES (PIDDAC), under the scope of the strategic funding of UIDB/04469/2020 (Centre of Biological Engineering, University of Minho) and UIDB/50006/2020 (Associated Laboratory for Green Chemistry - Clean Technologies and Processes, LAQV-REQUIMTE) unit, respectively.

## Acknowledgments

The authors thank all the peer reviewers who took time to review for this Research Topic.

## Conflict of interest

The authors declare that the research was conducted in the absence of any commercial or financial relationships that could be construed as a potential conflict of interest.

## Publisher’s note

All claims expressed in this article are solely those of the authors and do not necessarily represent those of their affiliated organizations, or those of the publisher, the editors and the reviewers. Any product that may be evaluated in this article, or claim that may be made by its manufacturer, is not guaranteed or endorsed by the publisher.

## References

[B1] Ferreira C. M. H. López-Rayo S. Lucena J. J. Soares E. V. Soares H. M. V. M. (2019a). Evaluation of the efficacy of two new biotechnological-based freeze-dried fertilizers for sustainable fe deficiency correction of soybean plants grown in calcareous soils. Front. Plant Sci. 10. doi: 10.3389/fpls.2019.01335 PMC6857624 31781134

[B2] Ferreira C. M. H. Soares H. M. V. M. Soares E. V. (2019b). Promising bacterial genera for agricultural practices: An insight on plant growth-promoting properties and microbial safety aspects. Sci. Total Environ. 682, 779–799. doi: 10.1016/j.scitotenv.2019.04.225 31146074

[B3] Petropoulos S. A. (2020). Practical applications of plant biostimulants in greenhouse vegetable crop production. Agronomy 10, 10–13. doi: 10.3390/agronomy10101569

[B4] Soares E. V. (2022). Perspective on the biotechnological production of bacterial siderophores and their use. Appl. Microbiol. Biotechnol. 106, 3985–4004. doi: 10.1007/s00253-022-11995-y 35672469

